# The use of FDG PET in the assessment of dementia syndromes: numbers from a reference center in Recife, Brazil

**DOI:** 10.1002/alz.093163

**Published:** 2025-01-09

**Authors:** Mariana Gonçalves Maciel Pinheiro, Alberto Henrique Torres Trindade da Silva, Luiz Eduardo Duarte Borges Nunes, Vitor Maia Arca, Milena Beatriz de Araújo Silva, Breno José Alencar Pires Barbosa

**Affiliations:** ^1^ Universidade Federal de Pernambuco, Recife Brazil

## Abstract

**Background:**

Positrons Emission Tomography associated with Computed Tomography – PET/CT using the 18F‐fluorodeoxyglucose is a well‐established exam for the context of dementia evaluation. However, FDG PET is a method still unavailable in most centers and diagnostic accuracy depends on the development of a flowchart that involves proper indication by clinicians and specialized evaluation by nuclear medicine specialists. Our study aims to investigate the indications of FDG PET in the differential diagnosis of dementia in a single center; secondarily, to evaluate how this method aided the diagnostic process.

**Method:**

single‐center, descriptive analysis of patients with an initial diagnosis of neuropsychiatric syndromes that underwent FDG PET between 2018‐2023. We used an Excel chart to register clinical data and diagnostic hypothesis, followed by the FDG PET results. The final classification of each patient’s clinical diagnosis and image diagnosis findings was: concordant, discordant and undetermined concordation.

**Result:**

56 patients were included. The mean age was 71 years‐old and 55.36% were female. The diagnostic impression of the PET‐CT report of these cases showed 13 non‐specific changes in the metabolic view, 16 changes compatible with Alzheimer’s Disease (AD), 7 Lewy Bodies Dementia (LBD), 11 Frontotemporal dementia (FTD), 1 was associated with Mild Cognitive Decline (CLD). Furthermore, 7 of the reports were described as “Negative for dementia” and 1 as progressive supranuclear palsy (PSP). The main indications for PET‐CT scans were suspicion between the diagnosis of AD versus FTD and AD versus LBD. Suspicions for pseudodementia and other diseases progressing to cognitive impairment were also evaluated. Of the 56 patients in the sample, 14 (25.00%) of the clinical impressions were discordant with the results of the imaging exam, 24 (42.86%) were concordant and in 18 (32.14%) comparisons the exam pointed to indeterminate result.

**Conclusion:**

In this study, we observed that PET‐CT had the contributive potential to diagnostics of dementia in the majority of analyzed cases, in which it added value to initial clinical suspicions. In this sense, expanding data and carrying out new studies is still necessary.

